# Energy Consumption Analysis of LPWAN Technologies and Lifetime Estimation for IoT Application

**DOI:** 10.3390/s20174794

**Published:** 2020-08-25

**Authors:** Ritesh Kumar Singh, Priyesh Pappinisseri Puluckul, Rafael Berkvens, Maarten Weyn

**Affiliations:** IDLab—Faculty of Applied Engineering, University of Antwerp—imec, Sint-Pietersvliet 7, 2000 Antwerp, Belgium; priyesh.pappinisseripuluckul@uantwerpen.be (P.P.P.); rafael.berkvens@uantwerpen.be (R.B.); maarten.weyn@uantwerpen.be (M.W.)

**Keywords:** LPWAN, LoRaWAN, DASH7, Sigfox, NB-IoT, energy efficiency, power consumption

## Abstract

The spectrum of Internet of Things (IoT) applications is exponentially growing, driving the demand for better energy performance metrics. In conjunction, Low Power Wide Area Networks (LPWAN) have evolved as long-range connectivity enabler with low management cost. The integration of LPWAN communication assists in reliable IoT operation with extended lifetime. Notable LPWAN technologies that contend for many of the IoT applications are LoRaWAN, DASH7, Sigfox, and NB-IoT. Most of the end-devices such as sensors and actuators are battery powered, therefore investigating energy consumption becomes crucial. To estimate the consumed power, it is important to analyze the energy consumption in wireless communication. This paper describes an empirical evaluation of energy consumption for LPWAN wireless technologies. We measure the current consumption of LoRaWAN, DASH7, Sigfox, and NB-IoT and derive the respective battery lifetime. These measurements help to quantify the energy performance of different protocols. We observe that LoRaWAN and DASH7 are more energy efficient when compared to Sigfox and NB-IoT. Finally, a case study on energy consumption is done on precision agriculture in the greenhouse, showing that battery lifetime in real applications can drop significantly from the ideal case. These results can be used for increasing the effectiveness of the IoT application by selecting the right technology and battery capacity.

## 1. Introduction

The Internet of Things (IoT), has gained a high momentum [1] in both industry and research community providing ease of exchange of data between application devices and sensors. The surge in the growth of IoT has shown a vast increase in the spectrum of applications like precision agriculture (PA), smart city, asset tracking, healthcare and many more [2]. Majority of these applications demands long range, low data rate with low deployment and management cost as an essential integrant. Thereby, short range radio technologies are comparatively not on high demand for high coverage applications. Wide range of data acquisition devices are already a part of IoT applications that significantly consider long range communication with low energy consumption to prolong the lifetime of the network without human intervention. Thereby, these demands have driven the emergence of Low Power Wide Area Networks (LPWANs) [3].

**Figure 1:**
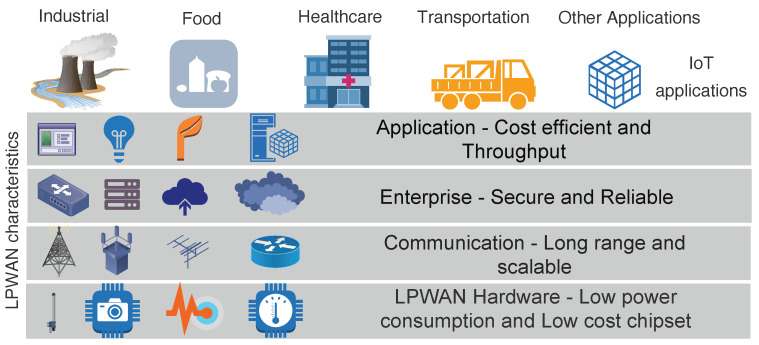
LPWAN characteristics that makes it an excellent choice for the IoT applications.

**Figure 2:**
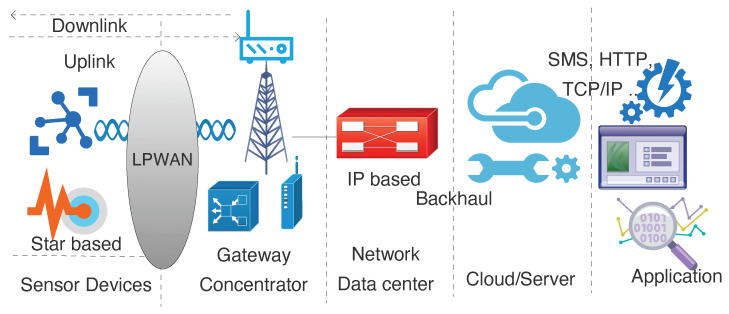
LPWAN common architecture featuring key elements and connectivity.

LPWAN is a category of wireless communication that acts as connectivity enabler to support diverse IoT applications by complimenting short and cellular wireless communication. Cellular based solutions have long connectivity but consume more energy [4]. LPWAN technology has got significant momentum [5], mainly because of its set of features like wide range, scalable deployment, highly energy efficient, and inexpensive in regards to the cost of management, operation, and radio chip-set. It provides the link range of one to multiple kilometers with support of connecting thousands of end-devices to the infrastructure (gateway) [5]. In this paper, we have used term end-device to denote the source of data transmitted over the network. Figure 1, shows the characteristics of LPWAN technology favouring the rise in number of IoT applications. It demonstrates the set of features at different layers as an added value for numerous applications. Many licensed and unlicensed LPWAN technologies have arisen, among which the most emergent technologies are LoRaWAN, DASH7, Sigfox, and NB-IoT [6]. They have a different set of configuration parameters to regulate and position the application. The common architecture for LPWAN technologies is shown in Figure 2, which comprises of sensor devices, gateway, core network and the application.

Most of the operating end-devices such as sensors and actuators are battery-operated, thereby power depletion becomes an imperative factor and needs an energy management approach to prolong the battery life. Monitoring of energy consumption is one of the crucial considerations to optimally design the network while fulfilling the application requirements. Each task that is responsible for sensing, processing, and packet transmission consumes energy which accumulates to the power consumption. Additionally, other factors also influence energy consumption that derives total lifetime of the end-device such as configuration parameters of respective LPWAN technology, type of the hardware, battery and the firmware used on the end-device. Therefore, it is crucial to investigate the characteristics of LPWAN energy consumption. However, most of the published works only focus on energy consumption to a limited extent, providing only rough estimates through simulation or investigating only one LPWAN technology. This opens up research questions: (1) Which LPWAN wireless technology is the best connectivity enabler in the context of energy consumption for the particular set of IoT applications? (2) What can be the estimated battery lifetime and how much mAh(Milliamp-hours) capacity of battery is needed for application? (3) Also, the trade-off between energy consumption calculation for the ideal case and real-time deployment.

To underpin the relevance of the research questions, the following are the contributions of this paper:

- Representation of the generic energy consumption states for LoRaWAN, DASH7, Sigfox, and NB-IoT end-device.
- Real-time power consumption analysis for transmitting the data and implication of different modes of LPWAN technology concerning energy performance.
- Investigation and analysis of the lifetime for LPWAN powered end-device with reference to the battery capacity.
- Real-time case study for energy consumption in an IoT application.

The remainder of this paper is structured as follows. Section 2, describes the state-of-the-art for energy consumption in LPWAN and, the evolution of energy-efficient approaches and its integration with LPWAN wireless technology. Section 3, demonstrates the generic LPWAN energy consumption states. Section 4 explains the approach for energy consumption along with in-depth energy consumption analysis for each communication technology. In Section 5, we discuss the important challenges impacting energy consumption and compared the battery life in accordance with different LPWAN technologies in Section 6. Section 7, provides the energy performance for a real-time use case. Finally, we discuss in Section 8 and Section 9 draws conclusions and outlines ideas for future work.

## 2. Related Work

Energy efficiency is the key requirement for any sensor based application. LPWAN technologies are relatively new and are used in both indoor and outdoor applications [7,8]. Several papers have explored and evaluated the energy consumption of different individual LPWAN technologies [9,10,11].

Driven by the challenges of energy depletion, many recent works have focused to increase the power efficiency of the network and reduce the power dissipation in communication. Mahmoud et al. [12], did a comparative study between different low power wireless technologies and demonstrate the choice of right protocol is vital for the battery life. Data rate and payload affect the power consumption, so it should be contemplated prior to selecting the protocol. Joseph et al. [13], proposed an approach for calculating energy effectiveness based on throughput and identification of energy-efficiency among existing cross over models. The paper on the comparative study of LPWAN technologies [5,8] shows that LoRaWAN and Sigfox are advantageous in terms of battery lifetime as compared with NB-IoT. Author Dali et al. [14], raises energy-efficiency as a challenge for LPWAN networks which demands better design techniques. Network parameters are the key pillar for the performance and the network lifetime. Paper [15], formulates and demonstrates the impact of network parameters on energy efficiency. In addition to the analysis, it states battery lifetime as a function of LPWAN transmission parameters. There are contributions in developing custom hardware for LPWAN [16] to optimize the key constraints of hardware design i.e cost and energy.

Many published works focus on technology-specific energy modeling and performance evaluation mainly on coverage, capacity, and energy consumption. The papers [11,17], present an analytical model to characterize the current composition, device lifetime, and cost of the delivery of each message. It also gives the theoretical lifetime of the battery while sending messages at a particular interval, maintaining the duty cycle. In the same context, Luis [9] did the modeling for the energy performance of LoRaWAN. Evaluation results from this paper claim to achieve one year lifetime with an appropriately configured LoRaWAN end-device. However, as LPWAN protocol are very similar to Aloha type so its energy performance needs better design scheme such as time synchronization [18,19]. Taoufik et al. [20] describes the energy consumption model estimating consumed power in each sensor element. LoRaWAN provides three different device classes, which has different performance and energy consumption. Cheong et al. [21] did the theoretical and experimental comparison of the LoRaWAN classes and proclaims the operational battery life of ten years with Class A device, considering that parameters are correctly configured. LoRaWAN is used for both indoor and outdoor monitoring. Paper [22], used LoRaWAN for indoor human-centric applications and did the power consumption to illustrate the importance of radio operation mode and the use of the adaptive data rate for power efficiency. Paper [10] presents an energy consumption model for NB-IoT devices using Power Saving Mode (PSM) and Extended Discontinuous Reception (eDRX), with a Poisson arrival process for uplink and downlink data transmissions. Sakshi et al. [23], surveyed on energy-efficient NB-IoT architecture and also proposed energy efficient approach for applications like agriculture and healthcare. In another tag collection application [24], the evaluation of DASH7 shows that it has better performance than slotted Aloha. For energy efficiency, application-specific parameters plays an crucial role and on top of it energy harvesting is done [25] to prolong the battery life. Most of the aforementioned published papers have done the technology specific evaluation and analysis. Thereby, it is vital to compare the LPWAN protocols and its battery consumption to estimate the lifetime and pick the right technology as per the application requirement.

## 3. Generic Energy Consumption Paradigm

**Figure 3:**
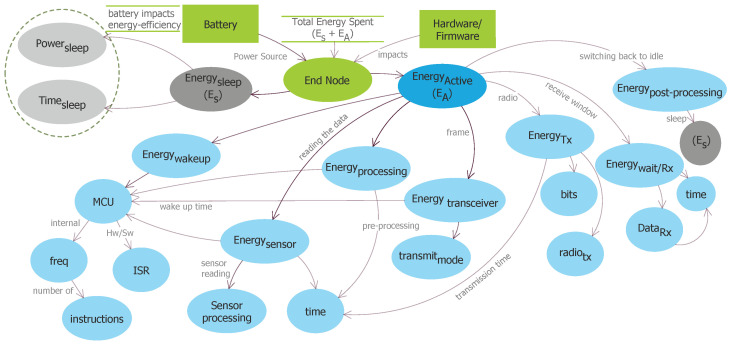
Generic representation for different states of energy consumption in an LPWAN end-device.

The energy consumption paradigm is discussed in this section and demonstrated using Figure 3. The autonomy of sensing and sending the data from a sensor node is a composite of divergent power consuming elements. In most of the sensor units, battery is the only source of power. Thereby, it is paramount to consider each element accountable for power consumption to calculate energy efficiency and its battery lifetime. The sensor unit has different operating modes imparting energy dissipation for the LPWAN based wireless communication. Broadly, this can be divided into two modes: *Non-active and Active period*. Non-active period constitutes of sleep and idle mode during which most of the peripherals of the sensor node are not performing any task. In the same context, the device moves to active mode where the processing happens, message packet is formed, and eventually data is transmitted using the LPWAN radio. The total energy spent by the end sensor node is the aggregated value of energy spent during sleep and energy spent during the active period. Energy spent during sleep is calculated by the total power consumed during the sleep period. Energy spent during an active period is the total energy spent during *Device wakeup* (warming up the micro-controller and initializing the end-device); *Sensor processing* (fetching the sensor value, this involves reading, parsing the sensor values and time involved for this activity); *Data processing* (processing the data and preparing packet frame); *Transceiver pre-processing* (transmitting the packet to transceiver which involves activating the radio for transmission mode); *Radio transmission* (utilizing radio for packet transmission, this depends upon LPWAN protocol, size of the payload and time-period for which radio is used); *Wait/Rx* (energy spent during the receive window or wait period to receive the acknowledgement); and lastly *post processing* (energy spent to stop all the aforementioned activity and going back to sleep again). Along with the energy consumed in the active period, the total energy spent is affected by three other major factors-battery used, firmware, and hardware. The impact of battery over the energy consumption by the end-device is discussed in Section 6. For each LPWAN protocol, there are a lot of hardware and firmware production partners with respective design detailing. Thus, the energy consumption may vary for each device. From a hardware perspective, the Printed Circuit Board (PCB) design, external hardwares such as Real Time Clock (RTC), internal frequency of the Micro-controller Unit (MCU), efficiency to handle number of instructions and other factors such as interrupt service routines impact the total energy consumption of the node. Different elements of the node active period depend upon the hardware efficiency such as processing of the sensor data and creating the packet for transmission. Also, on the other side firmware can alter the efficiency of handling the hardware by the inclusion of a timer, polling, software-based interrupts, and likewise approach. Thereby, it is essential to measure the energy consumption while excluding all the aforementioned factors to the nearest utilization point.

## 4. Approach, LPWAN Communication Technology, and Energy Consumption Analysis

In this section, we discuss the approach and assumptions used for calculating the energy consumption of the LPWAN technologies. We start with the approach, experimental setup, and then introduce the overview of different LPWAN technologies (LoRaWAN, DASH7, Sigfox and NB-IoT) in the context of both generic architecture and power consumption. Subsequently, the respective real-time energy consumption measurements are presented.

### 4.1. Device and Measurement Approach

To evaluate the energy consumption for LPWAN technology, firstly we adhere to Section 3 and secondly, to make the measurement consistent, we baselined the parameters and configurations across all measurements. The energy consumption in the end-device predominantly happens in sensor unit, processing unit and a communication unit. Following are the assumptions used in the experiment to evaluate the energy consumption.

- ensure constant MCU state among all end-devices;
- no external hardware such as RTC were connected with the modules;
- same battery type and capacity are used for all end-devices;
- fixed packet length of 5 bytes (no sensing is performed) with the provision of only uplink messages;
- techniques such as fog computing, edge computing, the local database are not considered but only real-time measurement and transmission is considered;
- end-devices are powered at the same voltage level equal to 3.7 V.

**Figure 4:**
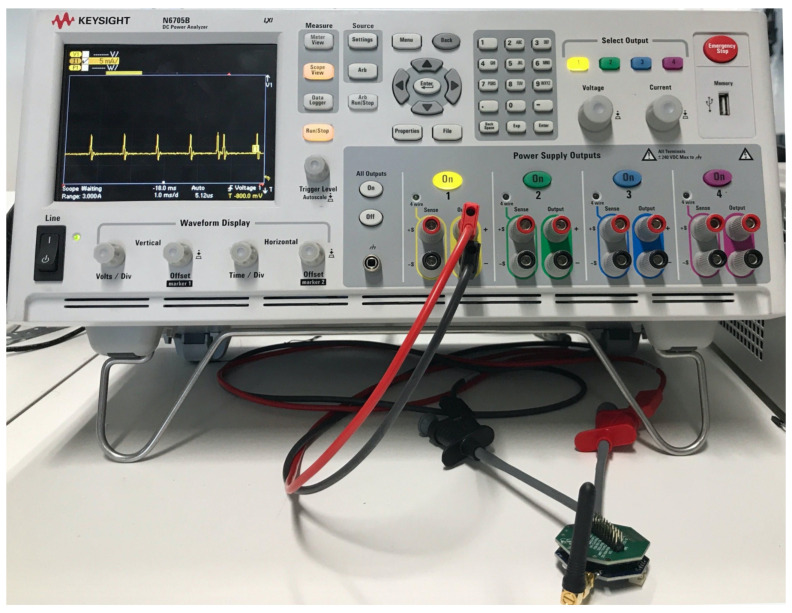
Experiment setup for energy measurement using Keysight N6705B DC Power Analyzer.

**Figure 5:**
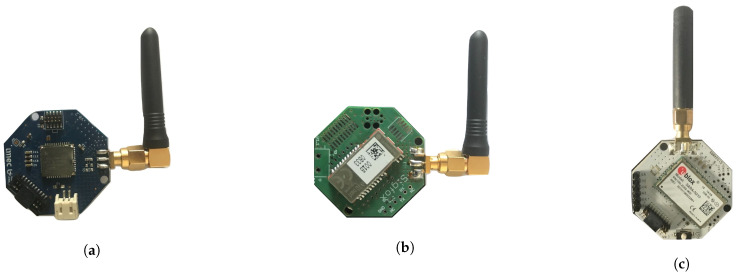
LPWAN modules used in the experiment for the energy analysis (**a**) LoRa/DASH7 module; (**b**) Sigfox module; (**c**) NB-IoT module.

For the energy measurements we have used two different power analyzers (to validate the results)-Keysight N6705B DC Power Analyzer [26] and Joulescope Precision DC Energy Analyzer [27]. The experimental setup is shown in Figure 4. It shows the setup of LPWAN end-devices connected to the power analyzer. The energy consumption data logs were further analyzed through the application software from the Keysight Technologies. We have used ABZ-078-860-930MHz LPWAN MCU Module [28] with Semtech (SX1276) and STM (STM32L) micro-controller for measuring LoRaWAN and DASH7, TD1207 module [29] for SigFox, and ultra-low power SARA-N2 module for NB-IoT [30] energy consumption as shown in Figure 5.

### 4.2. LPWAN Communication Technology

In this section, we discuss several characteristics and overview of the communication technology and perform the power consumption analysis.

#### 4.2.1. LoRaWAN

**Figure 6:**
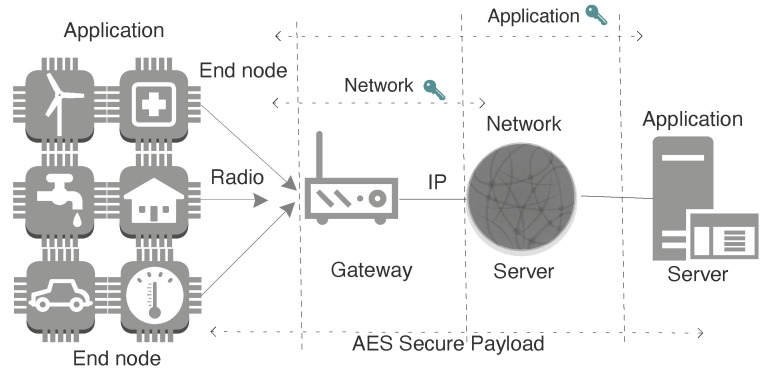
LoRaWAN network architecture.

LoRaWAN is an LPWAN communication technology designed to get long range, low cost, and low power star-of-stars topology [31]. LoRaWAN network constitutes of three basic elements: end-devices, gateway, and then a backhaul to the server as depicted in Figure 6. LoRaWAN provides security by incorporating end-to-end encryption using network and application key to maintain data integrity. End-device represents the sensors or actuators which uses LoRa physical layer to exchange message with the gateway [32]. Lora is a long range technology that uses the ISM band. This communication happens over the radio and further from the gateway to network server happens over IP based protocol stack. LoRa uses Chirp Spread Spectrum for low power and long-range transmission. LoRa radio provides an option for different configurations to align the requirement of the application. These parameters are *frequency* (region-based i.e., 863 MHz to 870 MHz in Europe); the *spreading factor* (SF i.e., number of chips per symbol between 6 and 12); *coding rate* (range between 1 to 4, where smaller rate has higher time on air); and the *bandwidth* (125 kHz, 250 kHz, or 500 kHz). Total derived energy consumption depends upon the combination of these parameters.

**Figure 7:**
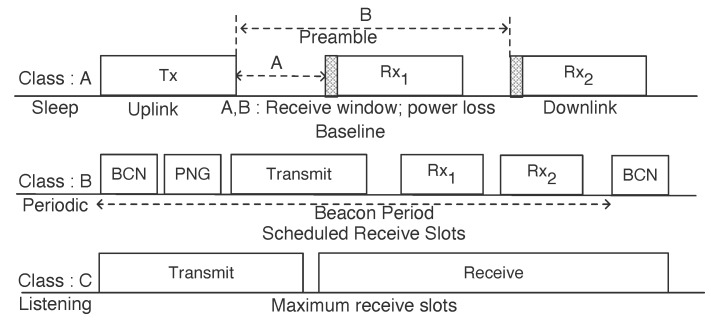
LoRaWAN device class.

Considering different set of applications, the LoRaWAN specification [32] has defined three classes of end-devices (A,B,C). These classes have different energy consumption and process of operation. Three modes of device classes and their functionality are illustrated in Figure 7 and explained as below:

- Class A: This is the most deployed class of end-device where the sensor can initiate uplink transmission as per the need and have to wait for two short downlink messages. This is the baseline class of device and most power efficient as compared to other class of devices. However, it uses a pure Aloha approach which results in considerable power loss. Most of the time this class of device sleeps to reduce energy consumption.
- Class B: In this device class, periodic ping slots are initiated by the gateway as downlink messages. This helps in giving schedule slots to the end-device for sending the uplink messages. However, due to periodic beacon from the gateway and its data rate, there is more power loss. End-device periodically receives one of the beacons from network to align internal clock with the network. This beacon based timing reference can be used by the end-device as receive window.
- Class C: This category of end-device consumes maximum power with almost continuous open receive windows. They offer lowest latency as it allows downlink transmission at any time. Mostly, this class of device is best suited for over the air firmware upgrade. Class C types of devices work same as Class A type, but they do not close the receive window until the next transmission.

**Figure 8:**
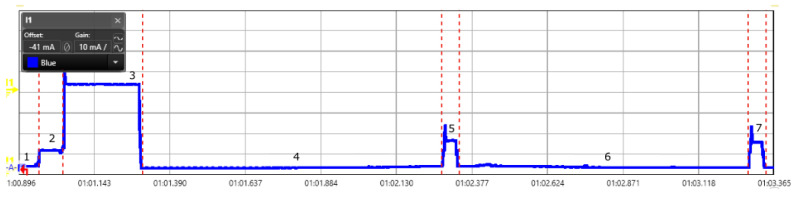
Scope view of a LoRaWAN message (Class A device) captured using power analyzer for the measurement of energy consumption.

We did the energy measurement for Class A type of devices as they are currently the most deployed device for several applications. We configured the end-device with SF 9. Different SF configuration results into varying on air time [33] and results into distinct energy consumption. The uplink message from a device is captured through the power analyzer and shown in Figure 8. State depicted in the Figure 8 represents different process of operations in a LoRaWAN message transmission. The measurement for current consumption, energy, and process time is shown in the Table 1. In Figure 8, state 1 exhibits the sleep mode; state 2 is the duration in which controller warms up and becomes active; state 3 is the transmission mode to send the message from end-device to the gateway; state 4 and 6 are the waiting period before opening first and second receive slot respectively; and state 5 and 7 are the receiving slots for the end-device where it waits to receive a message from the gateway.

#### 4.2.2. DASH7

**Figure 9:**
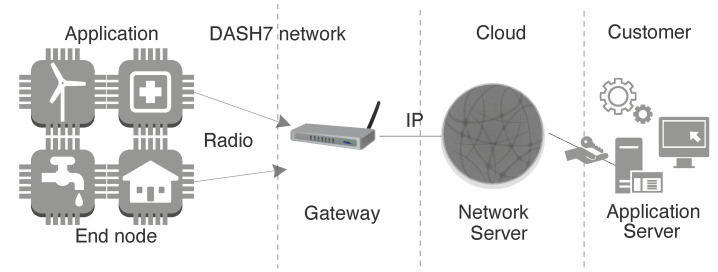
DASH7 network architecture.

DASH7 Alliance Protocol (D7A) [34] defines an ISM band operated communication protocol which is based on the ISO/IEC 18000-7 standard for RFID tags and sensor nodes. DASH7 is built around the concept of BLAST technology. *BLAST* stands for Bursty, Light, Asynchronous, Stealth, and Transitive. *Bursty* implies the abrupt and short transfer of data in response to an event or operation, which is the most likely requirement in low power wireless and actuator networks. *Light* refers to the small packet size which is usually limited to 256 bytes per transaction. *Asynchronous* is one of the major concepts in D7A standard; the transfer of commands and responses between the gateways and end-device can happen at any time. The end-devices are bound to the gateway and hence do not require any periodic beacons to advertise its presence and this is reflected in *Stealth*. Finally, *Transitive* implies the ability of the protocol to support mobility which allows the end nodes to move seamlessly across the gateways.The D7A supports 3 classes of device: the gateway, sub-controller, and endpoint. Out of the three, gateway and the endpoints are the widely implemented device classes. The end point is the least energy demanding device and implements only a minimal version of the protocol. End-device uses intermittent channel scanning to check for any incoming packet and spends most of its time in sleep. The sub-controller in essence, is an endpoint but it is a full featured DASH7 device. Gateway connects the low power network to another network and should implement all of the D7A stack functionalities. A fundamental structure of a DASH7 network is depicted in Figure 9.

**Figure 10:**
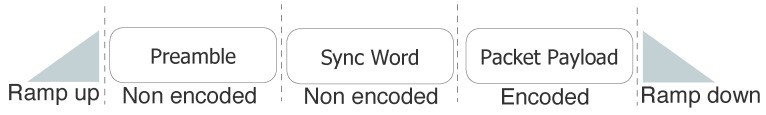
DASH7 message frame structure.

DASH7 physical layer specifies parameters like communication channels, modulation scheme, symbol encoding, and the packet structure. It uses Gaussian Frequency Shift Keying (GFSK) modulation scheme for modulating the data to be transmitted. Though, the specification mentions that there can be up to four modulation schemes, only two are implemented currently. The structure of the DASH7 frame is shown in Figure 10. Message packet comprises of preamble, sync word, and payload. Preamble defines a non-encoded binary string starting with ‘1’ and used for calibrating the receiver circuit. The specification recommends a minimum preamble length of 32 bits for LO_RATE(Low), NOR_RATE(normal) and 48 bit for HI_RATE(high) data rates. Sync word is a 16-bit string and is used to align the packet payload. The physical layer of DASH7 supports multiple sync words which can be used to differentiate the coding scheme employed. Along with these, a power ramp-up preceding and a ramp down following the packets are added to meet the band stop channel requirements.

The Data Link Layer (DLL) defines a background frame (carry the adhoc synchronization details) and a foreground frame (carry the normal payload). The length of the frames is variable and can have a maximum length of 256 bytes.

**Figure 11:**
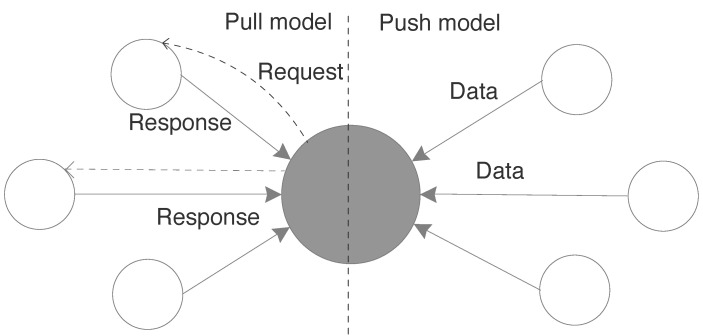
DASH7 communication model.

DASH7 supports two modes of communications- PULL mode and PUSH mode as shown in Figure 11. In PUSH mode, the end-device sleeps most of the time and wakes up at predefined time intervals to transfer or push data to the gateway. Whereas in PULL mode, the end-device wakes up intermittently to sniff the channel for any commands from the gateway. Thus gateway needs to pull the data from an endpoint, in contrast to push where the node voluntarily does the data dissemination. The concept of adhoc synchronisation is the key enabler of PULL mode communication. The adhoc synchronization lets the node to synchronize for commands from the gateway at any random time. When a gateway wants to read data from the end-device, it floods the channel with a background frame which contains a time value. This time value specifies the time when the gateway will send a foreground frame with the actual data. Any node receiving this background frame will go to sleep after switching on a timer with the previously received time value. When the timer triggers, the device will wake up and receive the foreground frame.

**Figure 12:**
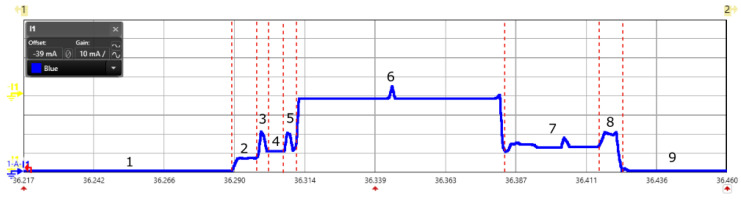
Scope view of a DASH7 PUSH mode captured using power analyzer for the measurement of energy consumption.

We profiled the energy consumption of DASH7 in both the PUSH and PULL modes and are presented in Table 2, Table 3 and Table 4. The end-devices were configured to transmit in LOW_RATE mode with transmission power +14 dBm. The current consumption in PUSH mode as captured by the power analyzer is shown in Figure 12. State 1 and 9 in the shown scope view represents the low power sleep mode of the device. DASH7 uses polite spectrum access and hence performs Clear Channel Assessment (CCA) before transmitting a packet. Current consumed in CCA operation is shown by states 3 and 5. Once CCA succeeds, end-device enters transmission mode, which is state 6 to disseminate the packet. Once the transmission is done the end-device enters state 7 where it waits for an Acknowledgement (ACK) from the gateway. Finally, after receiving an ACK in state 8, it goes back to sleep mode. The reception of an ACK is optional and happens if the end-device has explicitly requested for an ACK from the gateway.

**Figure 13:**
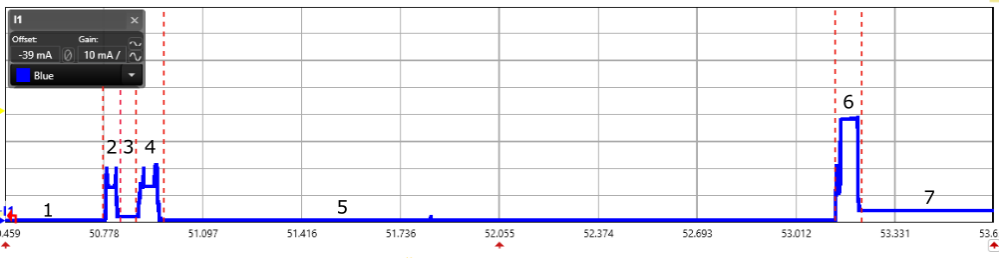
Scope view of a DASH7 PULL mode with background frame, foreground frame and data transmission captured using power analyzer for the measurement of energy consumption.

**Figure 14:**
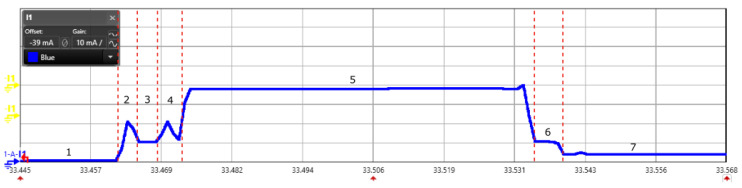
Scope view of a DASH7 data transmission in PULL mode captured using power analyzer for the measurement of energy consumption.

The PULL mode of data transmission is shown in Figure 13 and Figure 14. It follows a similar current consumption pattern except that there are periodic peaks caused by radio wake-ups. Figure 13 shows a background, foreground, and data transmission process. Where as, Figure 14 shows a detailed view of a packet transmission. In one of the wake-up periods, a background frame is received by the node which is marked by state 2. The background frame contains the time period of state 3 in which the device go backs to low power mode. The reception of a foreground frame happens in state 4 and in state 6 the node transmits the data to the gateway.

#### 4.2.3. Sigfox

**Figure 15:**
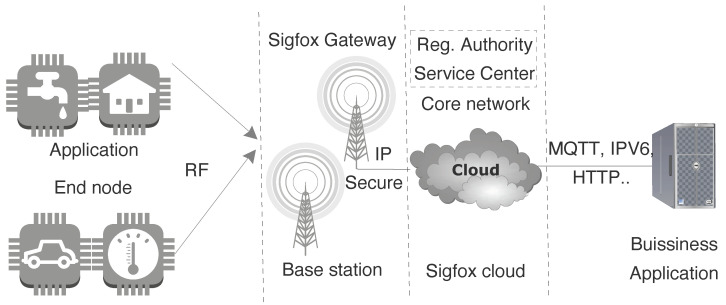
Sigfox network architecture.

The basic elements of the Sigfox network are end-devices, base stations, and back-end core network as shown in Figure 15. Sensors connect to the neighboring base station using wireless connection and then through the public network to the core network as per specification [35]. Since end-device can connect to any base station which helps in supporting mobility, it avoids handover procedures. Service Center (SC) and the Registration Authority (RA) are an integral part of the core network. They help to manage the base station, end-device, and authorizing the network access from security point of view. Business or applications at the other end have the capability to interact with the end-devices via user interface by using the provided Application Program Interfaces (APIs). Sigfox supports unidirectional and bidirectional communication using Ultra Narrow Band (UNB) radio, in the bands 868.00 MHz–868.60 MHz and 869.40 MHz to 869.65 MHz (Europe region) for uplink and downlink transmission respectively. The bandwidth for the uplink is region-specific with a maximum transmit power of 25 mW in Europe region however, for downlink channel it is 1.5 kHz. Sigfox uses license-free spectrum and different modulation schemes i.e Differential Binary Phase-Shift Keying (DBPSK) and GFSK for uplink and downlink respectively.

**Figure 16:**
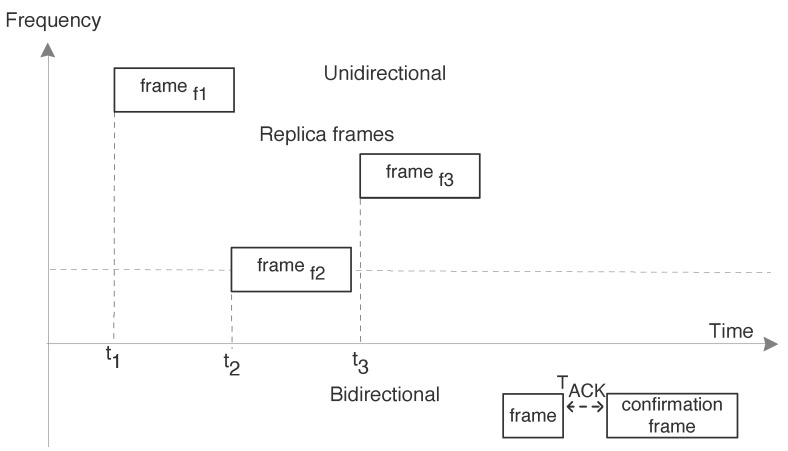
Sigfox unidirectional and bidirectional message transmission with replica messages at different frequency.

**Figure 17:**
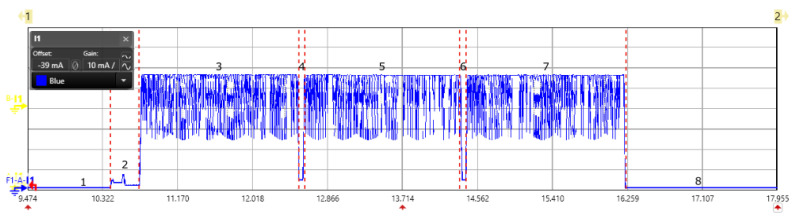
Scope view of a Sigfox data transmission captured using power analyzer for the measurement of energy consumption.

The end-device initiates the connection in an asynchronous manner and for rest of the time it stays in sleep to save the energy consumption. The communication from end-device to the base station enables cooperative reception as the message is received by multiple base stations empowering spatial diversity. In the unidirectional communication, end-device transmits the uplink message over random frequency channel followed by its two replica messages using other channels at different time intervals as shown in Figure 16. On one side, this increases communication robustness and guarantee of packet delivery up to a certain extent but on other side consumes more power. These transmissions are unconfirmed as as there is no response from base station. Sigfox does not support retransmissions in case the acknowledgment is not received from the base station. In bidirectional transactions, message is transmitted in the same manner as stated above for unidirectional messages but after completing transmission, end-device opens up a receive window to enable reception of a downlink message from the base station. We have calculated the energy consumption for a unidirectional packet transmission from the end-device as depicted in Figure 17. The analysis of the power consumption for different process in the communication such as current, time, and energy consumed are presented in the Table 5. The states in the Figure 17 demonstrates the different process involved in communication such as: state 1,8 is the sleep mode; state 2 is the phase where device warms up and activates the module; state 3 is the packet transmission for the first message; state 4,6 is the wait time before sending the replica message; and state 5,7 are the transmission of the replica message for the first original message.

#### 4.2.4. NB-IoT

**Figure 18:**
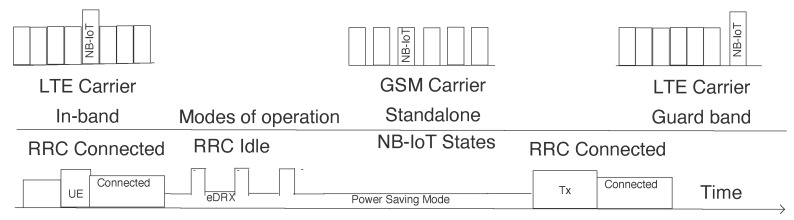
NB-IoT states and modes of operation.

NB-IoT is a narrowband LPWAN technology under licensed frequency bands and uses the existing LTE deployed infrastructure [36]. It exists together with LTE or GSM with a frequency bandwidth of 200 KHz. NB-IoT supports different operation modes as: *Stand alone operation* (utilization of GSM frequency bands); *Guard band operation* (utilization of LTE carrier’s guard-band that are unused); and *In-band operation* (utilization of resource blocks of LTE carrier) as depicted in Figure 18. NB-IoT is a miniature form of the LTE protocol as it reduces the functionalities and enhances as per IoT requirement. It uses LTE physical and higher protocol layers by using LTE backend system. It uses QPSK and FDMA for uplink and OFDMA for downlink transmission with maximum throughput rate of 20 kbps and 200 kbps respectively. The maximum payload size for one message can be 1600 bytes. NB-IoT has power-saving states as shown in the Figure 18.

The Radio Resource Control (RRC), is responsible for the mechanism of setup, modification and to release resources. It got two states: connected and idle state. All the communication happens in the RRC connected state. However, it consumes more energy, so once the communication is over the end-device moves back to RRC idle state, which triggers after network specific inactivity timer is over. RRC idle is constituted of two modes: Extended Discontinuous Reception Mode (eDRX) and Power Saving Mode (PSM) mode. From energy consumption point of view, eDRX is sleep mode which can vary from 0 to 186 min and PSM is deep sleep mode which can be up to up to 413.3 days. The eDRX mode consists of paging time window which is between 2.56 and 40.96 s. Initially, the end-device registers in the network and goes in a connected state. Thereafter, it enters the eDRX state where it can listen to the downlink control channel (Narrowband Physical Downlink Control Channel). This listening is termed as paging which ends when the end-device moves to PSM state. In this state, end-device keeps it registration state but turns off its radio to save energy.

**Figure 19:**
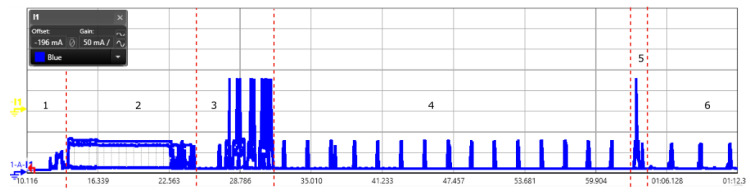
Scope view of a NB-IoT data transmission captured using power analyzer showing all the states.

**Figure 20:**
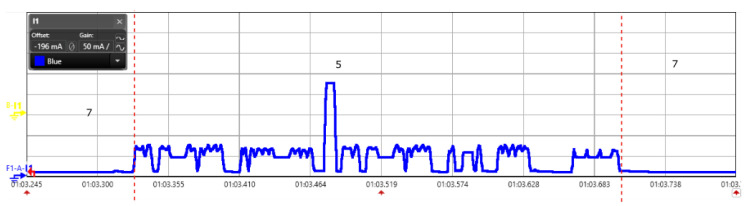
Scope view of a NB-IoT data transmission and deep sleep.

We have done the power consumption for the NB-IoT end-device as shown in Figure 19. It starts with the device activation and initialization state marked as state 1; then starts the registration process with the network service provider as shown in state 2. This state is specific to the network provider and is of 10 s. In our case, we had Orange as network service provider. In the subsequent state 3, we did the communication using AT commands to open a socket for the communication. State 4,6, is the eDRX state where the node does the paging to listen any downlink message. The eDRX mode can be configured to save the energy consumption. The timer value impacts the overall energy consumption and it is aligned with the application requirement. The state 5 is the transmission mode to send the message. The zoomed in scope view for the message transmission is shown in Figure 20. State 7 represents the PSM mode, where the radio gets switched off and becomes unavailable. Once the PSM state ends, the next state is the transmission state. The current consumption for each state is shown in the Table 6, to send one message. For our experiment, we used the AT commands (AT commands manual from SARA-N2 / SARA-N3 series) to add the device to the network. Post registration, we opened a new UDP socket for sending UDP data to a server. Following which sending a fixed payload of 5 bytes. The device had deep sleep mode enabled and the messages were sent at an interval of five minutes.

## 5. Potential Challenges in Estimating Energy Efficiency For End-Device

Estimating the actual energy consumption of a low power end-device exhibits many challenges in real-life scenarios. There are many factors which determine the overall energy consumption of the end-device. A slight variation in these parameters can have a significant impact on the energy consumption of the end-device; for instance, the sleep mode current. As seen from the current consumption of different LPWAN technologies, the sleep mode current has a significant impact on the over all energy consumption of the device. As most of the time, end-device is in an in-active state so it should be optimized to achieve energy-efficiency.

Battery characteristics also impact the end-device lifetime [37] and task oriented power consumption. There has been research from a quantitative standpoint to predict and model the battery lifetime. The complexity is mainly due to the nonlinear battery discharge because of factors such as battery electrode, rate of diffusion of active materials in electrolyte, etc. Apart from the energy consumption required in the process of computation and communication from the end-device; aspects such as chemical changes, thermal effects and discharge rate of the battery also impact the battery lifetime. Also, the inevitable leakage current from electronics components that results from the temperature variation can lead to an uncertainty in determining the actual energy consumption of a device. This is of more importance in the case of outdoor devices where the temperature variation can be significant. As a result, the dormant mode energy draw of the device from the battery is dependent on both the hardware and the environmental conditions where the device is installed.

Firmware and hardware are the backbone for the end-device and directly contributes in energy consumption. With each new release of LPWAN commodity hardware implementations, they are expected to optimize the power consumption which brings the added value for the deployment. There are techniques to reduce power consumption by writing better software [38]. Efficient implementation of firmware in microcontroller helps in efficiently managing the resources. There has to be careful coordination between hardware and software for the critical role of initializing and configuring modules, controlling the task and other power management features. There are several LPWAN hardwares and batteries available in the market so it depends on the application to pick the right choice in contrast to, aforementioned factors responsible in energy consumption.

Applications with multiple integrated devices need far more than battery management, such as keeping the pack of battery cells healthy. Interfacing the battery load needs a safe operation. There are multiple sensors operating at different voltage and speed involved in the operation of such devices. This generates varying workloads and heat on the battery which impacts the expected lifetime. This involves multi-directional research scope such as protecting batteries from cyber attacks using wireless networks and accurate battery management. Energy management is one of the biggest challenges in portable applications having heterogeneous components with dynamic processing. There are various intelligent energy management systems such as fuzzy-logic-approach to reduce energy consumption. Thereby, the type of application and its complexity has an impact in estimating energy efficiency. This needs to be evaluated for picking the right battery, its capacity, and wireless technology for the smooth working of the application. Moreover, applications that has an autonomous power source such as an energy harvesting system can have an extended algorithm and energy management technique to map the number of message transmissions to the available onboard energy. There is a trade-off between available energy and the application requirement that can be achieved by managing the frequency of message transmissions and optimal management of the computation.

## 6. Battery Life Estimation

**Figure 21:**
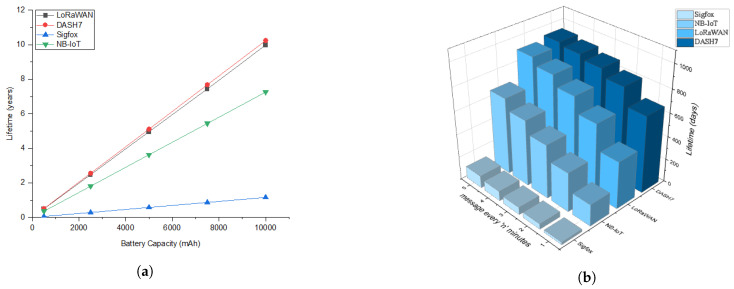
Lifetime estimation for different LPWAN technologies: (**a**) Different battery capacities; (**b**) Different frequency of message transmission (every 1,2,3,4,5 min with constant 2500 mAh battery).

Having prior knowledge of device lifetime when operated from a battery can be beneficial during the design phases of the network. Battery selection can then be made to meet the device lifetime requirement. Based on the data produced in the energy consumption analysis, we estimated a theoretical lifetime for all the four LPWAN modules for different battery capacity. A plot of the estimated life and battery capacity is shown in Figure 21a. The graph is intended to assist a developer to decide the battery capacity for a particular communication protocol and device lifetime. However, it must be noted that the data presented are assuming the ideal operating conditions. Factors like temperature and humidity can have an impact on battery capacity and the leakage current of the electronics used as discussed in Section 5. Moreover, we have considered only the radio transmission and reception processes, and no other activities are considered for the lifetime estimation. Nevertheless, one should be able to estimate the approximate actual life time by adding the energy consumption of the other required states to the base model. An example calculation for LoRa is listed in Table 7. We notice that LoRaWAN and DASH7 have got better energy consumption performance with extended lifetime compared to NB-IoT and Sigfox. We also analyzed the trade-off in lifetime calculation with variation of message frequency while keeping the battery capacity constant (2500 mAh) as shown in Figure 21b. This can be useful to look over the different lifetime of the end-device while sending message every 1,2,3,4 and 5 min respectively.

### 6.1. System Regulations and Duty Cycle

Each frequency band used by the LPWAN technologies imposes limit to the number of transmissions. These limits are defined as duty cycle and polite spectrum access restrictions [39]. Duty cycle is measured as ratio of total transmissions per observation period (default period is one hour). The range of duty cycle is between 0.1% to 10% which is the transmission time in the utilized frequency band. It helps in avoiding the congestion of the network. The battery life depends upon number of transmissions, while following the region specific duty cycle. There are no distinctions on the even spacing between messages but only maximum duty cycle needs to be respected. The number of transmissions depends on the application requirement such as alarm system, transportation or smart home. So, the total battery life estimation is proportional to the maximum limit of the duty cycle and the message transmissions required by the application. Few applications use polite spectrum access encompassing listen before talk and adaptive frequency agility. In this case, duty cycle restrictions are loosened and device uses channel assessment check to identify if medium is already in use and accordingly wait or change the frequency. This results into additional complexity and impacts the overall power consumption by the end-device.

### 6.2. Transmission Power Limitations

In addition to the limitations imposed by the duty cycle, there is a limit on the transmission power which affects the total battery life. Transmission power is expressed in milliwatt (mW) or decibel-milliwatt (dBm) that ranges from 5 to 2000 mW. For example in Europe, Sigfox has a duty cycle of 1% and maximum output power of 25 mW [40]. For each LPWAN technology there is a configuration setting for power adaptation which results into different energy consumption. The power configuration is tightly coupled with the distance of end-device from the gateway. Most of the LPWAN technologies operating in the ISM band has a power setting of 14dBm whereas transmission power in cellular are around 20 dBm. For example, LoRaWAN end-device has the option to configure the data rate and transmit power or use the Adaptive Data Rate (ADR) mechanism that is managed by the network server. The paper [41], demonstrates the impact of LoRa transmission parameter selection on communication performance and energy consumption. Also, it shows the significance of transmission settings to satisfy performance requirements. Thereby, for calculating the battery life it is vital to consider the configuration parameters for the end-device, need for retransmissions caused due to collisions, and scenario where acknowledgement is expected by the end-device.

## 7. Real-Time Application Use-Case

**Figure 22:**
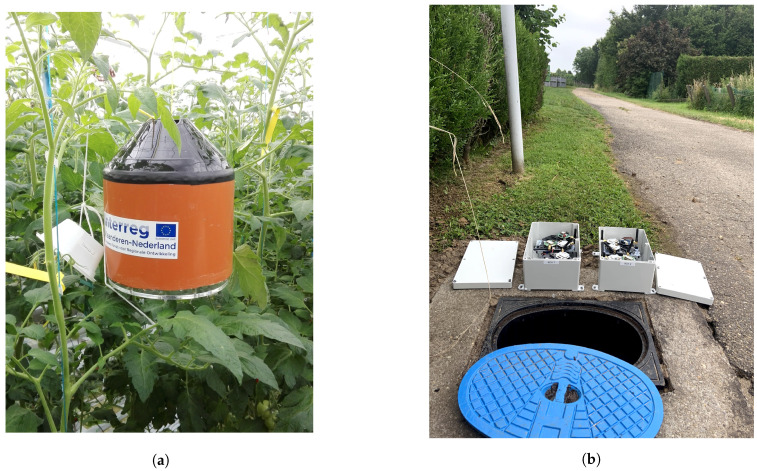
(**a**) Deployment of LoRaWAN based sensor network at the greenhouse; (**b**) Sensors deployment in remote locations i.e., underground water grid.

In Section 4, we calculated the energy consumption of different LPWAN technologies in an ideal environment. However, in real time measurement there are several factors that impact the energy consumption as mentioned in Section 5. We deployed a LoRaWAN based network in a greenhouse [42] based out in Belgium to monitor the environment as shown in Figure 22a. In this setup, we used imec’s OCTA-Connect platform board which has onboard temperature and humidity sensors. It is mounted with LoRaWAN shield for the communication. The sensor device measures the sensing values after each five minutes and forwards the message to the gateway. The LoRaWAN Kerlink gateway is installed inside the greenhouse with an ethernet and power connection. It receives the message from the installed sensors and forwards the data to the things-network. In most of the applications, it is very difficult to change the batteries or charge them during the deployed phase. This is at times avoided to maintain the security and sanity of the devices or the deployment is at the remote location. For example, Figure 22a shows the deployment of a sensor box in a tomato greenhouse where hygiene is of utmost priority to avoid any plant disease. Same way, Figure 22b shows the sensor setup that is deployed to locate the water leakage across the water grid. This deployment is mostly spread across outskirts and remote location which makes the battery replacement a strenuous task.

The energy consumption of the end-device differs from idle case to the state in deployment due to factors such as: sensors attached to the end-device, exposure to the environment, extra time in computation, and behavior of battery. For this deployment, in addition to the idle energy consumption of LoRaWAN message there is additional task for taking the measurement from the sensors and processing the message frame. Also, it was observed that the temperature inside the sensor box increases which affects the overall energy consumption due to the generated heat. The current consumption for the sensing setup (Figure 22a), is shown in Table 8. There is some time and energy invested in reading sensor values. As per the length of the message and protocol-dependent configuration settings the radio transmission consumes energy. In our case, its LoRaWAN data rate, spreading factor and power configurations. We have used SF 9 for our experiments and exhausted a Li-Ion battery of 15,000 mAh capacity for 60,480 messages in approximately 7 months. This is much less when compared with the calculations presented in Table 1. In the ideal case, the device should have lasted for approximately 10.9 years with a packet transmitted in every 5 min. This behaviour of the device in real time can be traced back to a hardware and software imperfections in the device design which increased the inactive (idle) mode energy consumption of the device to 6 mA. Where the end-device used for the empirical evaluation in Section 4 has a inactive mode current of 80 μ A. Along with this, the shelf time and self discharging of the batteries could have contributed to the deterioration in the over all life time of the device.

## 8. Discussion

LPWAN technologies are limited by system regulation of the duty-cycle, which restricts the number of message transmission. The energy consumption analysis helps to calculate the energy requirement in different phases but its important to include other factors such as the battery, hardware, and firmware to calculate the end-device lifetime. Energy consumption depends on the type of hardware picked and the attached peripherals such as sensors. There are elements such as LEDs, resistors, and likewise part of the PCB design that consumes additional energy. For our experiments, we removed such connections to make it close to the ideal setup for better comparison. *LoRaWAN* has class A type of device that is most power efficient as compared to other classes of devices. However, we observed that after each transmission there is a waiting period for two receive windows which consumes energy. This pure Aloha design scheme can be solved by using a better approach such as time synchronization or co-ordination scheme to make it efficient and qualify for multi-hop communication. *DASH7* is different than LoRaWAN in the context of range and it does the polite channel access before it sends. The optimized utilization of PUSH and PULL mode helps to qualify for numerous applications while saving energy. On top of the existing solution design scheme such as waking up radio using a preamble based approach and using better channel coding scheme can help further in energy optimization. *Sigfox* has an advantage over other only from coverage perspective, otherwise, it has the highest energy consumption. It sends two more replica of a message in addition to the original message to support mobility and packet loss. This increases the radio transmission time and leads to spending more energy. Acknowledgement based approach or scheduling among end-devices can help the Sigfox network to extend the battery life. *NB-IoT* is the most robust technology among all due to its availability, coverage, and throughput. The end-device spends initial time in registration and communicating with network which is fixed from the service provider; optimization of this period can save energy. Also, the right selection of eDRX and PSM mode can help the application to have a prolonged lifetime. In our experiments, we observed that LoRaWAN and DASH7 have better energy performance as compared to NB-IoT and Sigfox. The most crucial part which impacts the overall energy consumption is the sleep or the idle time. As the end-device spends the maximum period in this state so it should consume the minimal energy consumption. Most of the research paper, claims the ideal energy consumption using the LPWAN commodity hardware but it turns upside down on the real-time deployment. As we saw, that in ideal case we got the LoRaWAN lifetime for several years but with the real time deployment it narrowed down to a few months. Thereby, its important to note this trade-off and estimate the lifetime of the LPWAN based network on the basis of environment, sensors, reading interval, and firmware.

## 9. Conclusions

Energy consumption is one of the most crucial requirements for design and implementation of an IoT application. In this paper, we have analysed the energy consumption and quantified the energy performance of the LPWAN technologies. Also, we calculated the battery lifetime for different technologies in respect to the divergent battery capacity. Results show that message transmission and current consumption of idle period is the main contributor for the overall battery lifetime. Based on the analysis of our experiment results, it can be concluded that LoRaWAN and DASH7 are more energy-efficient as compare to NB-IoT and Sigfox. However, LoRaWAN attracts application with better coverage distance and DASH7 covers the application space demanding mid range communication. Further, NB-IoT is more efficient than other technologies where availability is of utmost importance. Sigfox has the lowest energy consumption performance as compared to other LPWAN technologies. We demonstrated the lifetime of LPWAN technologies with different battery capacity and message transmission frequency. This can give insights and help in picking the right LPWAN technology, managing the frequency of communication, and finally selecting the appropriate battery capacity.

IoT requires LPWAN technology and battery powered sensors to be compatible with the lifetime demand of the application. In ideal case, the battery lifetime of the LPWAN network stretches over several years, as it is multiple years in our evaluation. However, as shown later in the paper for the real-time case study on greenhouse deployment, the battery life decreases drastically. This gives three level of energy-consumption analysis: first level with simulation, second with the real-time hardwares, and finally the real time analysis of battery life post deployment. As we proceed in the above sequential analysis, a more realistic energy consumption evaluation can be achieved that can improve the future designs. Our future work will focus on the evaluation of the impact of LPWAN configuration parameters towards energy efficiency and also to propose a feedback model for calculating the lifetime of the network prior to actual deployment.

## Figures and Tables

**Table 1:** Current composition split into different states of operation for LoRaWAN.

State	Process	Time (ms)	Avg. Current (mA)	Energy (mJ)
1	Sleep	variable	0.081	variable
2	Controller active	80.28	7.79	2.31
3	Tx window	255.89	39.14	37.05
4	Rx wait time 1	993.48	0.081	0.29
5	Rx window 1	50.17	11.24	2.08
6	Rx wait time 2	951.33	0.081	0.28
7	Rx window 2	50.17	10.49	1.94

**Table 2:** Current composition split into different states of operation for DASH7 PUSH mode.

State	Process	Time (ms)	Avg. Current (mA)	Energy (mJ)
1,9	Sleep	Variable	0.1	Variable
2	Device wakeup	8.028	5.66	0.16
3	CCA1	4.014	13.60	0.20
4	Wait between CCA	3.011	10.11	0.11
5	CCA2	4.014	14.77	0.21
6	Packet transmission	72.25	37.21	9.94
7	Wait for ACK	33.11	12.50	1.53
8	ACK received	9.03	16.21	0.54

**Table 3:** Current composition split into different states of operation for DASH7 background and foreground scanning.

State	Process	Time (ms)	Avg. Current (mA)	Energy (mJ)
1,5	Sleep	variable	0.10	variable
2	Background frame(bg)	37.13	12.26	1.68
3	Wait between (bg-fg)	69.24	1.38	0.35
4	Foreground frame (fg)	67.23	12.49	3.10
6	Radio Packet	75.14	31.05	8.64

**Table 4:** Current composition split into different states of operation for DASH7 PULL mode.

State	Process	Time (ms)	Avg. Current (mA)	Energy (mJ)
1	Sleep	variable	0.10	variable
2	CCA1	4.01	10.01	0.14
3	Wait for CCA2	5.94	9.94	0.21
4	CCA2	3.09	15.96	0.17
5	Radio Tx	59.12	36.54	7.99
6	Controller activity	3.40	7.24	0.091

**Table 5:** Current composition split into different states of operation for Sigfox.

State	Process	Time (ms)	Avg. Current (mA)	Energy (mJ)
1,8	Sleep	variable	0.0065	variable
2	Module active	316.60	2.06	2.46
3	Tx 1	1798	48.32	321.45
4,6	Wait time	48.03	3.78	0.67
5	Tx 2	1795	49.50	328.75
7	Tx 3	1803	49.57	330.68

**Table 6:** Current composition split into different states of operation for NB-IoT.

State	Process	Time (ms)	Avg. Current (mA)	Energy (mJ)
1	Initialization	5390	8.46	167.52
2	Module activation with service provider	11,030	57.63	2326.22
3	Connection	6930	28.07	717.94
4,6	eDRX	31,000	9.58	1089.65
5	Tx mode	373	46.12	63.48
7	deep sleep	variable	0.10	variable

**Table 7:** Battery life estimation for LoRaWAN end-device.

Process	Value
Transmission interval	300 s
Number of packets per day	288
Battery Capacity	133,200 J (10,000 mAh)
Energy spent in one transmission	39.73 mJ
Total energy spent in 288 transmission per day	11.44 J
Current consumption in sleep mode	0.08 mA
Energy spent in sleep in a day	25.37 J
Total Energy spent in a day	36.82 J
Expected device life time	9.9 Years

**Table 8:** Power consumption analysis for LoRaWAN message used for the Greenhouse monitoring application.

Phase	Time	Avg. Current (mA)
Sensor reading	82.28 ms	13.24
Radio transmission	249.8 ms	46.08
Inactive mode	5 min	6.01
One total message	2.3 s	10.92
